# Supplementary material for: Capturing the primordial Kras mutation initiating urethane carcinogenesis
Source: Nat Commun. 2020 Apr 14;11:1800. doi: 10.1038/s41467-020-15660-8 (PMC7156420; doi:10.1038/s41467-020-15660-8)
Supplement: Supplementary file 1 — Supplementary Information [file 41467_2020_15660_MOESM1_ESM.pdf]

# Supplementary Information

## Capturing the primordial *Kras* mutation initiating urethane carcinogenesis

Li et al.

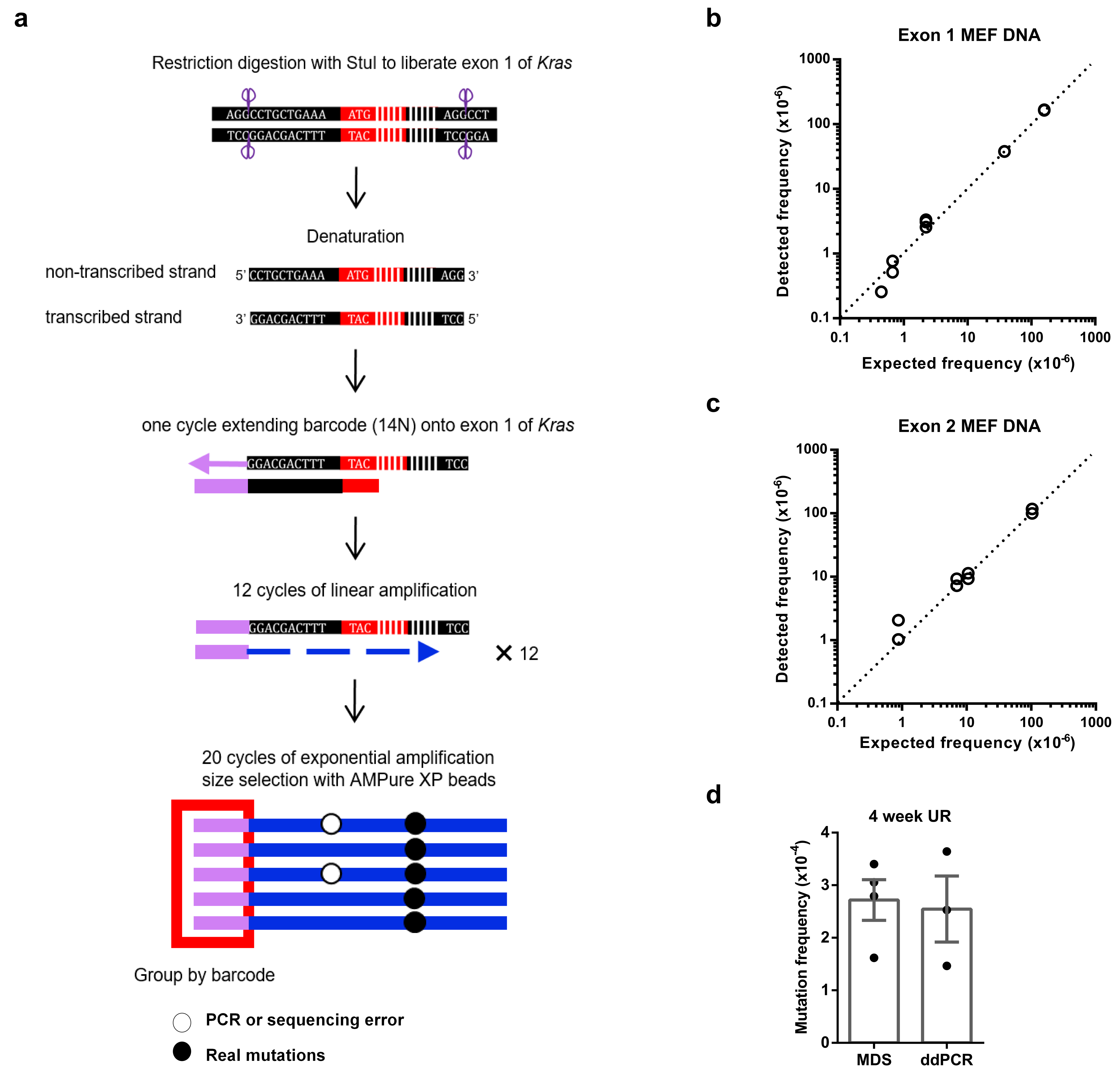


**Supplementary Fig. 1 | MDS optimized to detect ultra-rare mutation in the mammalian genome. a,** Diagram of MDS assay optimized for the transcribed strand of exon 1 of *Kras* based on Ref 13. **b,c,** Frequency of single (detected) versus co-occurring (present) mutations identified by MDS using a dilution series of *Kras* cDNAs with 2-3 different mutations engineered in **b,** exon 1 or **c,** exon 2 mixed with genomic DNA from MEFs. **d,** Mean ± SEM frequency of *Kras*$^{Q61L}$ mutations detected in the lungs of mice 4 weeks after exposure to urethane (UR) by MDS targeting the non-transcribed strand of exon 2 of *Kras* versus droplet digital PCR (ddPCR) (n=4 mice for MDS and 3 mice for ddPCR from one experiment).

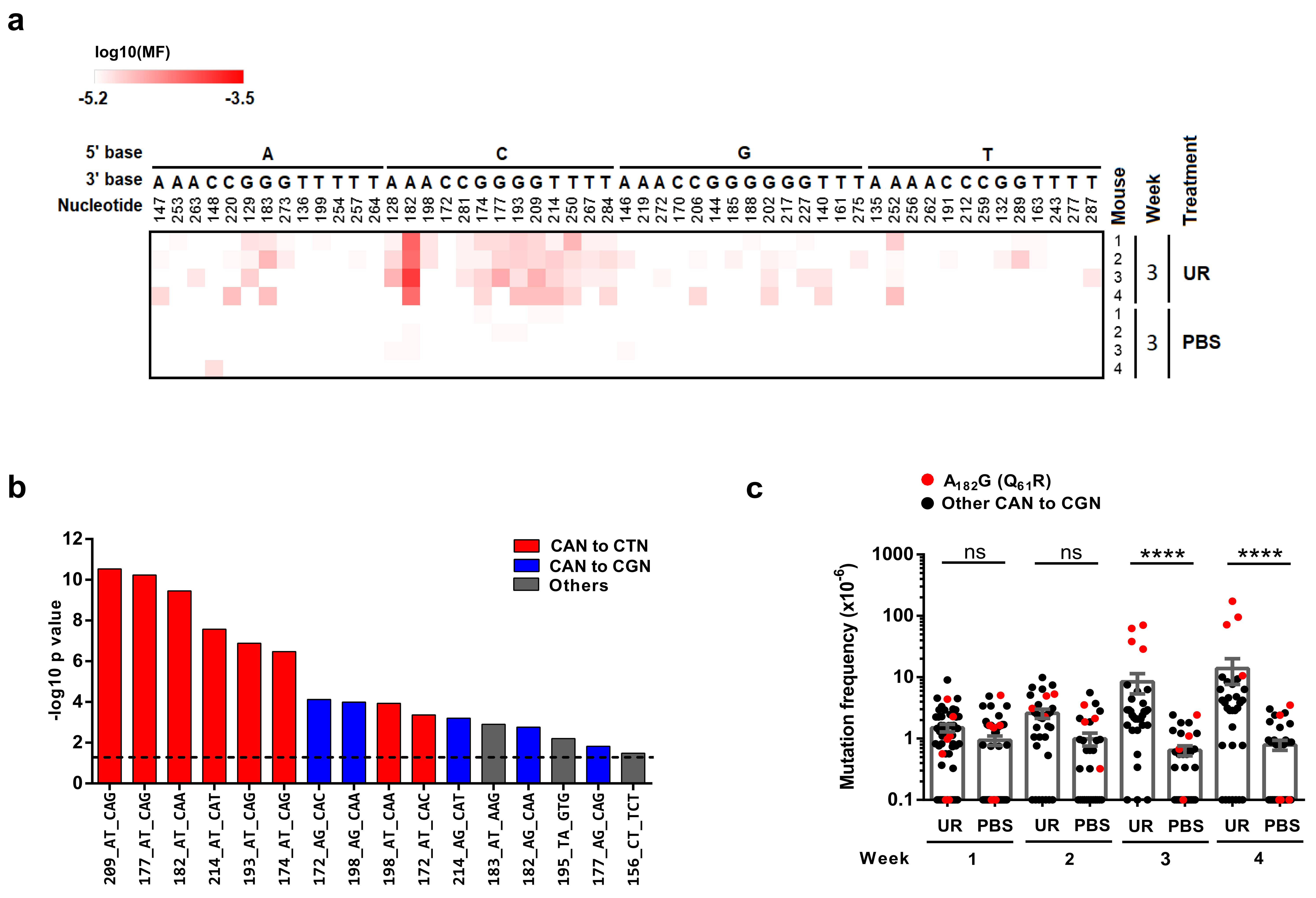


**Supplementary Fig. 2 | MDS detects urethane mutation specificity.** **a,** Heatmap of the mutation frequency (MF) determined by MDS for the transcribed strand of exon 2 of *Kras* from the lungs of mice 3 weeks after exposure to urethane (UR) or PBS (n=4 mice from one experiment), plotted as log transformed versus each A>T transversions (nucleotide number as well as the 5' and 3' base of the substituted A are shown at the top). **b,** Log10 *p* value of substitutions (shown as nucleotide position_substitution_trinucleotide) identified by MDS targeting the non-transcribed strand of *Kras* exon 2 that are significantly increased in mice exposed to urethane (UR) compared to PBS (Fig. 1, n=19 mice for UR and 20 mice for PBS from one experiment). *p* value calculated by two-tailed Mann-Whitney U test comparing all samples from urethane-exposed mice with all samples from PBS-exposed mice from 1 to 4 weeks. Dotted line: $p = 0.05$. **c,** Mean ± SEM mutation frequency of each possible CAN to CGN transitions at the indicated time points after mice were exposed to urethane (UR) or PBS (Fig. 1, n=7 mice at 1 week and 4 mice at 4 week from one experiment). *p* values calculated by Dunn's multiple comparison test following Kruskal-Wallis test. ****$p<0.0001$ and ns: not significant.

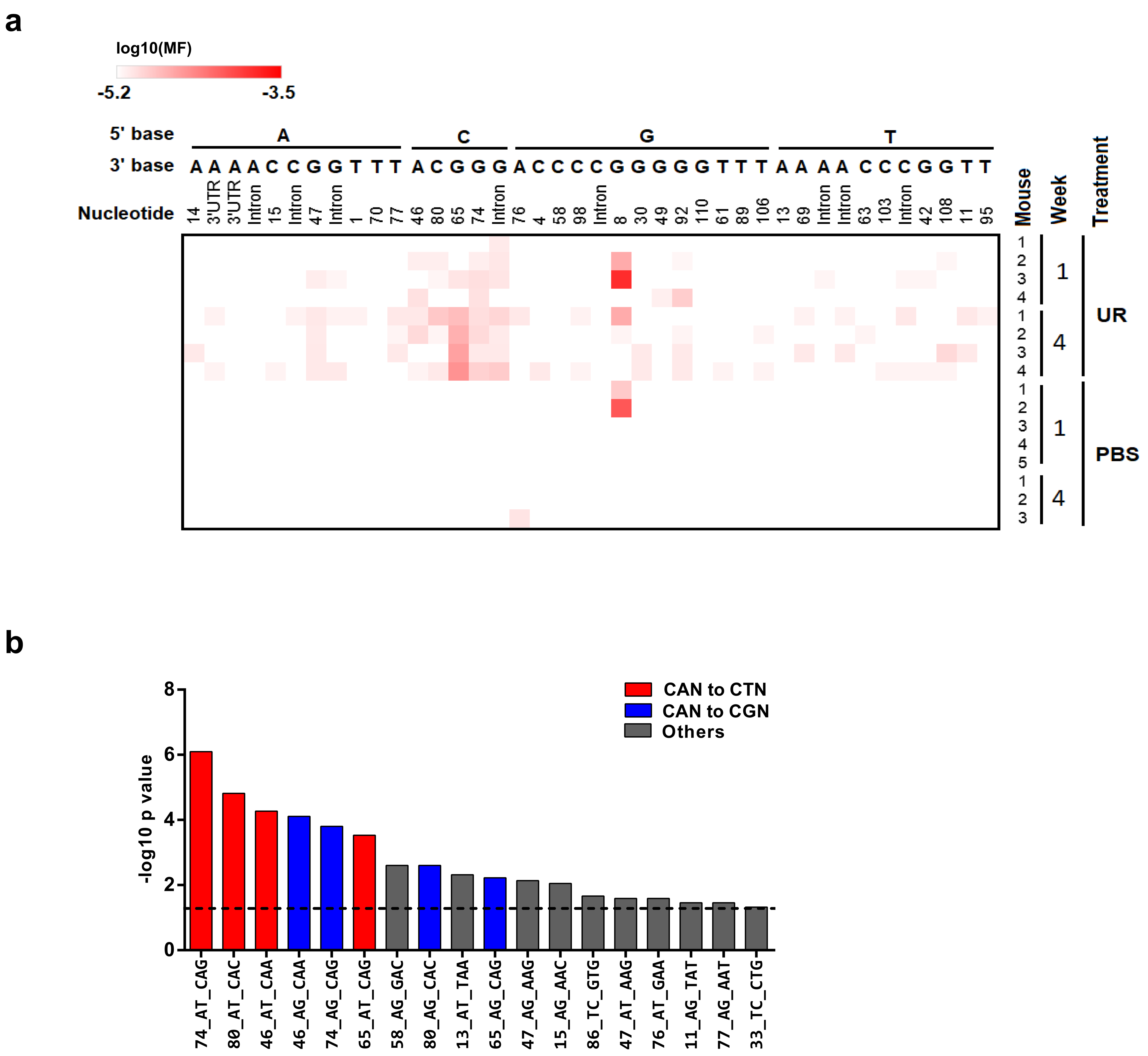


**Supplementary Fig. 3 | MDS detects the substitution tropism of urethane. a,** Heatmap of the mutation frequency (MF) determined by MDS for the non-transcribed strand of exon 1 of *Kras* from the lungs of mice at the indicated time points after exposure to urethane (UR) or PBS (n=4 mice for UR at 1 and 4 week, 5 mice for PBS at 1 week, and 3 mice for PBS at 4 week from one experiment), plotted as log transformed versus each A>T transversions (nucleotide number as well as the 5' and 3' base of the substituted A are shown at the top). **b,** Log10 *p* value of substitutions (shown as nucleotide position_substitution_trinucleotide) identified by MDS targeting the transcribed strand of *Kras* exon 1 that are significantly increased in mice exposed to urethane (UR) compared to PBS (Fig. 2, n=13 mice for UR and 11 mice for PBS from one experiment). *p* value calculated by two-tailed Mann-Whitney U test comparing all samples from urethane-exposed mice with all samples from PBS-exposed mice from 1 to 4 weeks. Dotted line: $p = 0.05$.

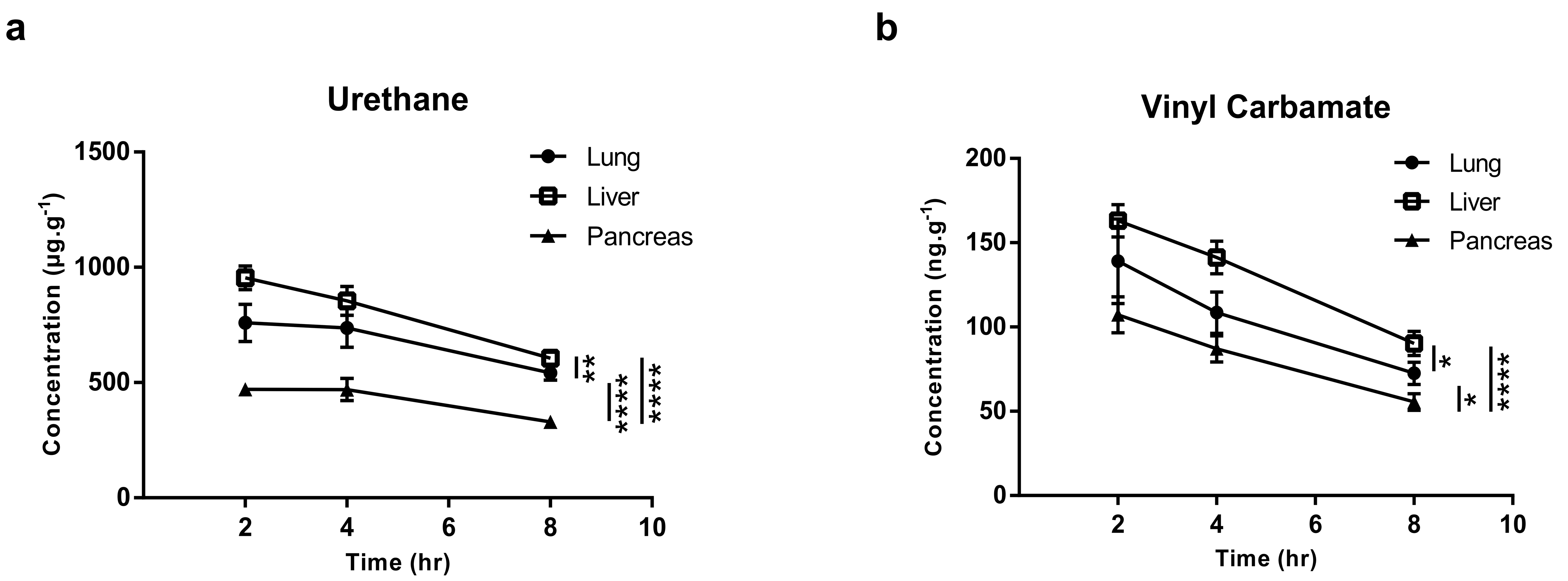


**Supplementary Fig. 4 | Distribution of urethane and its metabolite across tissues.** Mean ± SEM concentration of **a,** urethane or **b,** vinyl carbamate at the indicate time points after mice were injected with urethane in indicated tissues determined by LC/MS/MS (n=4 mice from one experiment). *p* values calculated by Holm-Sidak multiple comparisons test following two-way ANOVA. ****$p$<0.0001, **$p$<0.01, and *$p$<0.05.

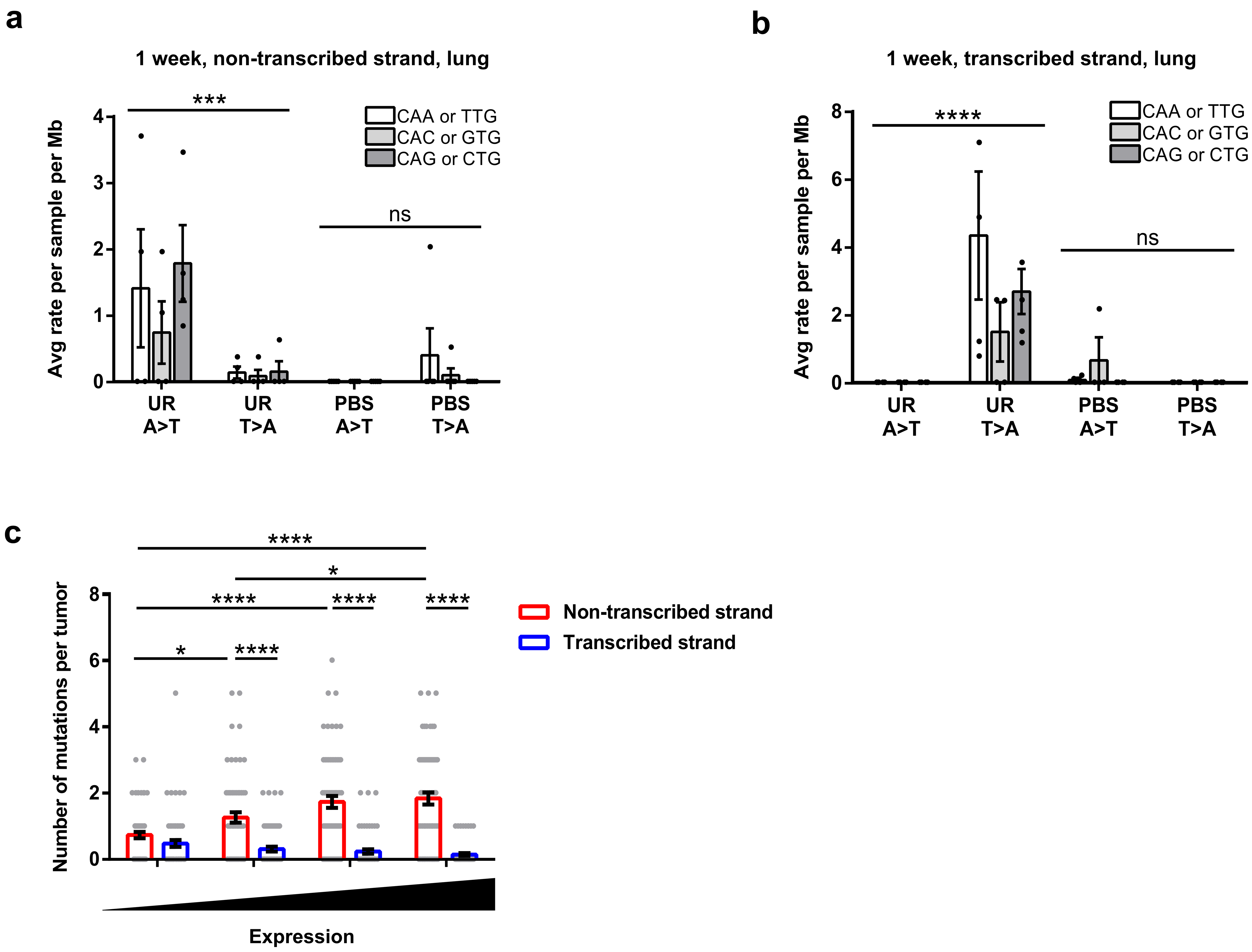


**Supplemental Figure 5 | MDS detects urethane strand bias. a,b,** Mean ± SEM mutation frequency of the indicated CAN to CTN transversions and reverse-complementary substitutions averaged by nucleotide positions determined by MDS sequencing for *Kras* exon 1 **a,** non-transcribed (n=4 mice for UR and 5 mice for PBS from one experiment) or **b,** the transcribed strand strand (Fig. 2, n=4 mice from one experiment) from the lungs of mice 1 week after exposure to urethane (UR) or PBS. **c,** Mean ± SEM frequency of CAN to CTN transversions of the non-transcribed versus transcribed strand in urethane-induced tumors from whole exome sequencing data[3] in genes binned by their mRNA levels from the mouse lung reported in the mouse ENCODE project[37] (n=66 tumors). *p* values calculated by Holm-Sidak multiple comparisons test following two-way ANOVA. ****$p$<0.0001, ***$p$<0.001, *$p$<0.05, and ns: not significant.

**Supplementary Table 1. Effect of sequencing depth on barcode recovery efficiency.**

| Sample | Raw reads ($x10^6$) | Raw reads / number of input cells | Barcodes recovered ($x10^6$) | Barcodes with R > 1 ($x10^6$) | Barcodes with R = 1 (%) |
|---|---|---|---|---|---|
| 1 | 2.1 | 0.5 | 1 | 0.5 | 47 |
| 2 | 3.2 | 0.8 | 0.6 | 0.4 | 23.5 |
| 3 | 4 | 1.0 | 1.1 | 0.7 | 32.4 |
| 4 | 4.6 | 1.2 | 1 | 0.6 | 33.2 |
| 5 | 4.9 | 1.3 | 1.3 | 0.9 | 28.9 |
| 6 | 6.4 | 1.6 | 1.1 | 0.9 | 25.3 |
| 7 | 8.4 | 2.2 | 1.4 | 1.1 | 22.7 |
| 8 | 8.7 | 2.2 | 1.1 | 0.9 | 19.4 |

R: number of independent reads sharing the same barcode

**Supplementary Table 2. Frequency of spiked-in mutations in MEF or murine lung genomic DNA.**

| Source of genomic DNA | Target exon | Mutant clone | Mutation | Frequency of mutant present | Frequency of mutant detected |
|---|---|---|---|---|---|
| MEF | Exon 1 | Clone 3 | A69T | 6.69E-07 | 5.13E-07 |
| | | | A74G | 6.69E-07 | 7.70E-07 |
| | | Clone 4 | A63T | 4.46E-07 | 2.57E-07 |
| | | | C73G | 4.46E-07 | 2.57E-07 |
| | | Clone 6 | G50A | 2.23E-06 | 3.08E-06 |
| | | | T56A | 2.23E-06 | 2.57E-06 |
| | | | A77G | 2.23E-06 | 3.34E-06 |
| | | Clone 10 | T62C | 3.79E-05 | 3.80E-05 |
| | | | A74T | 3.79E-05 | 3.80E-05 |
| | | Clone 12 | T6A | 0.000159 | 0.000164266 |
| | | | A61T | 0.000159 | 0.000166319 |
| | Exon 2 | Clone 1 | T155A | 8.82E-07 | 1.03E-06 |
| | | | T232C | 8.82E-07 | 2.06E-06 |
| | | Clone 5 | T141A | 0 | 0 |
| | | | G226T | 0 | 1.03E-06 |
| | | Clone 6 | T155C | 0 | 0 |
| | | | T234C | 0 | 1.03E-06 |
| | | Clone 8 | A182G | 8.82E-07 | 1.03E-06 |
| | | | T215C | 8.82E-07 | 1.03E-06 |
| | | | A219G | 8.82E-07 | 2.06E-06 |
| | | Clone 9 | C168A | 1.06E-05 | 9.26E-06 |
| | | | G229T | 1.06E-05 | 1.13E-05 |
| | | Clone 11 | A148G | 7.06E-06 | 7.20E-06 |
| | | | C235T | 7.06E-06 | 9.26E-06 |
| | | Clone 12 | A135G | 0.000103 | 9.98E-05 |
| | | | C150T | 0.000103 | 0.000117319 |
| | | | G247A | 0.000103 | 0.000117319 |
| Lung | Exon 1 | Clone 3 | A69T | 3.30E-07 | 3.76E-07 |
| | | | A74G | 3.30E-07 | 3.76E-07 |
| | | Clone 4 | A63T | 6.59E-07 | 1.13E-06 |
| | | | C73G | 6.59E-07 | 7.51E-07 |
| | | Clone 6 | G50A | 3.30E-06 | 4.13E-06 |
| | | | T56A | 3.30E-06 | 3.00E-06 |
| | | | A77G | 3.30E-06 | 2.63E-06 |
| | | Clone 10 | T62C | 4.95E-05 | 4.09E-05 |
| | | | A74T | 4.95E-05 | 4.09E-05 |
| | | Clone 12 | T6A | 0.000218 | 0.000199025 |
| | | | A61T | 0.000218 | 0.000197147 |
