# Supplementary material for: Capturing the primordial Kras mutation initiating urethane carcinogenesis
Source: Nat Commun. 2020 Apr 14;11:1800. doi: 10.1038/s41467-020-15660-8 (PMC7156420; doi:10.1038/s41467-020-15660-8)
Supplement: Supplementary file 3 — Description of Additional Supplementary Files [file 41467_2020_15660_MOESM3_ESM.pdf]

Description of Additional Supplementary Files

File Name: Supplementary Data 1
Description: Barcode recovery and MDS primers for all samples.

File Name: Supplementary Data 2
Description: Frequency of mutations in Kras exon 2 detected by MDS targeting the non-transcribed strand in the lungs of mice after PBS or urethane exposure.

File Name: Supplementary Data 3
Description: Frequency of mutations in Kras exon 2 detected by MDS targeting the transcribed strand in the lungs of mice after PBS or urethane exposure.

File Name: Supplementary Data 4
Description: Frequency of mutations in Kras exon 1 detected by MDS targeting the transcribed strand in the lungs of mice after PBS or urethane exposure.

File Name: Supplementary Data 5
Description: Frequency of mutations in Kras exon 1 detected by MDS targeting the non-transcribed strand in the lungs of mice after PBS or urethane exposure.

File Name: Supplementary Data 6
Description: Frequency of mutations in Hras exon 2 detected by MDS targeting the non-transcribed strand in the lungs of mice after PBS or urethane exposure.

File Name: Supplementary Data 7
Description: Frequency of mutations in Kras exon 2 detected by MDS targeting the non-transcribed strand in the liver of mice after PBS or urethane exposure.

File Name: Supplementary Data 8
Description: Frequency of mutations in Kras exon 2 detected by MDS targeting the non-transcribed strand in the pancreas of mice after PBS or urethane exposure.
