# Supplementary material for: Capturing the primordial Kras mutation initiating urethane carcinogenesis
Source: Nat Commun. 2020 Apr 14;11:1800. doi: 10.1038/s41467-020-15660-8 (PMC7156420; doi:10.1038/s41467-020-15660-8)
Supplement: Supplementary file 12 — Reporting Summary [file 41467_2020_15660_MOESM12_ESM.pdf]

nature research

Corresponding author(s): Christopher M. Counter

Last updated by author(s): Mar 10, 2020

Reporting Summary

Nature Research wishes to improve the reproducibility of the work that we publish. This form provides structure for consistency and transparency in reporting. For further information on Nature Research policies, see Authors & Referees and the Editorial Policy Checklist .

Statistics

For all statistical analyses, confirm that the following items are present in the figure legend, table legend, main text, or Methods section.

n/a	Confirmed	
☐	☒	The exact sample size (*n*) for each experimental group/condition, given as a discrete number and unit of measurement
☐	☒	A statement on whether measurements were taken from distinct samples or whether the same sample was measured repeatedly
☐	☒	The statistical test(s) used AND whether they are one- or two-sided<br>*Only common tests should be described solely by name; describe more complex techniques in the Methods section.*
☒	☐	A description of all covariates tested
☐	☒	A description of any assumptions or corrections, such as tests of normality and adjustment for multiple comparisons
☐	☒	A full description of the statistical parameters including central tendency (e.g. means) or other basic estimates (e.g. regression coefficient) AND variation (e.g. standard deviation) or associated estimates of uncertainty (e.g. confidence intervals)
☐	☒	For null hypothesis testing, the test statistic (e.g. *F*, *t*, *r*) with confidence intervals, effect sizes, degrees of freedom and *P* value noted<br>*Give P values as exact values whenever suitable.*
☒	☐	For Bayesian analysis, information on the choice of priors and Markov chain Monte Carlo settings
☒	☐	For hierarchical and complex designs, identification of the appropriate level for tests and full reporting of outcomes
☒	☐	Estimates of effect sizes (e.g. Cohen's *d*, Pearson's *r*), indicating how they were calculated

Our web collection on statistics for biologists contains articles on many of the points above.

Software and code

Policy information about availability of computer code

Data collection	No software was used for data collection.
Data analysis	The following software tools are publicly available and used as described in METHOD:<br>Galaxy web platform (https://galaxyproject.org)<br>PEAR pair-end read merger (http://www.exelixis-lab.org/web/software/pear)<br>Morpheus (https://software.broadinstitute.org/morpheus)<br>GraphPad Prism 6<br>Real Statistics Resource Pack (www.real-statistics.com)

For manuscripts utilizing custom algorithms or software that are central to the research but not yet described in published literature, software must be made available to editors/reviewers. We strongly encourage code deposition in a community repository (e.g. GitHub). See the Nature Research guidelines for submitting code & software for further information.

Data

Policy information about availability of data

All manuscripts must include a data availability statement. This statement should provide the following information, where applicable:
- Accession codes, unique identifiers, or web links for publicly available datasets
- A list of figures that have associated raw data
- A description of any restrictions on data availability

All raw Illumina® sequencing data has been deposited to NCBI Sequence Read Archive (SRA) under accession number PRJNA561927. Additional data generated in this study are available within the paper and in the supplementary information. Publicly available datasets for mutations detected in pulmonary tumors and gene expression in mouse lung are accessible from the cited original publications.

Field-specific reporting

Please select the one below that is the best fit for your research. If you are not sure, read the appropriate sections before making your selection.

- [x] Life sciences
- [ ] Behavioural & social sciences
- [ ] Ecological, evolutionary & environmental sciences

For a reference copy of the document with all sections, see nature.com/documents/nr-reporting-summary-flat.pdf

Life sciences study design

All studies must disclose on these points even when the disclosure is negative.

Sample size	Statistical methods to predetermine sample size were not used. Sample sizes were based on previous studies from others.
Data exclusions	For the analysis of sequencing data, samples with less than 150,000 barcode families passing filter (see METHOD) were excluded in order to maintain the depth required for mutant recovery.
Replication	The main findings of the paper were highly-reproducible among independent biological replicates. The number of independent biological replicates is indicated in each Figure Legend.
Randomization	Mice cohorts were selected to be approximately half males and half females.
Blinding	Blinding was not relevant to this study as all samples were processed with molecular biological techniques and analyzed through the same absolute quantitative method without human subjectivity.

Reporting for specific materials, systems and methods

We require information from authors about some types of materials, experimental systems and methods used in many studies. Here, indicate whether each material, system or method listed is relevant to your study. If you are not sure if a list item applies to your research, read the appropriate section before selecting a response.

Materials & experimental systems

n/a	Involved in the study	
x		Antibodies
	x	Eukaryotic cell lines
x		Palaeontology
	x	Animals and other organisms
x		Human research participants
x		Clinical data

Methods

n/a	Involved in the study	
x		ChIP-seq
x		Flow cytometry
x		MRI-based neuroimaging

Eukaryotic cell lines

Policy information about cell lines

Cell line source(s)	Mouse embryonic fibroblast derived from Kras wild-type mouse
Authentication	Presence of wild-type murine Kras was confirmed with PCR.
Mycoplasma contamination	The cell lines were not tested for mycoplasma contamination. This is not expected to impact our study since we used the MEFs solely as a source of genomic DNA.
Commonly misidentified lines (See ICLAC register)	No commonly misidentified cell lines were used.

Animals and other organisms

Policy information about studies involving animals; ARRIVE guidelines recommended for reporting animal research

Laboratory animals	6-8 week old male and female A/J mice (Jackson Laboratories, catalog number 000646) were used for experiments.
Wild animals	No wild animals were used.
Field-collected samples	No field-collected samples were used.

Ethics oversight | All mouse care and experiments were performed in accordance with protocols approved by the IACUC of Duke University.

Note that full information on the approval of the study protocol must also be provided in the manuscript.
